# Seasonal Dietary Changes Drive Adaptive Responses in the Gut Microbiota and Function of *Myotis chinensis*

**DOI:** 10.1002/ece3.73281

**Published:** 2026-03-17

**Authors:** Ruohan Xiong, Jing Li, Ke Wang, Hongying Kong, Zhengyu Dai, Yanni Wang, Rongquan Zheng

**Affiliations:** ^1^ College of Life Sciences Zhejiang Normal University Jinhua China; ^2^ Key Lab of Wildlife Biotechnology, Conservation and Utilization of Zhejiang Province Zhejiang Normal University Jinhua China

**Keywords:** dietary variations, functional prediction, gut microbiota, high‐throughput sequencing, *Myotis chinensis*

## Abstract

Gut microbiota play a crucial regulatory role in host energy metabolism and environmental adaptation, with their composition and function significantly influenced by dietary shifts. This study employed high‐throughput sequencing to analyze the dietary composition of *Myotis chinensis* and the seasonal dynamics of its gut microbiota, with functional predictions used to explore adaptive changes in metabolic pathways. Results indicated notable seasonal variations in dietary composition: Lepidoptera constituted the majority in spring (60.13%), and the diet transitioned to Orthoptera dominance in early summer (78.19%), with Odonata becoming the leading prey in late summer (79.99%), and Arachnida (47.93%) became the dominant taxon in autumn. Gut microbial diversity reached its maximum in late summer and was at its minimum during autumn. Functional predictions indicated significant upregulation of carbohydrate and amino acid metabolism pathways in summer, while lipid metabolism and hibernation‐related pathways were enriched in autumn. These synergistic dietary and microbial functional adjustments help *Myotis chinensis* adapt to seasonal energy demands and climatic extremes. This study provided novel insights into the ecological adaptation mechanisms of insectivorous bats.

## Graphical abstract

This research clarifies the synergistic adaptive interactions between seasonal foraging strategies and gut microbiome composition in Chinese chiropterans. Microbial diversity and community structure exhibit seasonal fluctuations, with functional metabolic pathways aligning to host reproductive cycles and hibernation physiology, thereby substantiating relevant hypotheses.

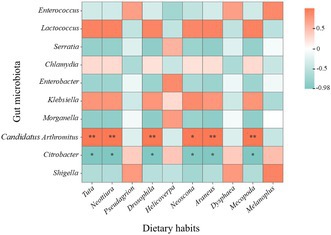

## 1 Introduction

Insectivorous bats constitute a critical functional group in ecosystems, regulating insect prey populations and contributing to ecological stability. Their seasonal dietary shifts represent a key adaptive strategy to cope with fluctuating food resources. The gut microbiome, acting as a vital metabolic interface, is intricately linked to these dietary changes and host environmental adaptation through its roles in nutrient processing, energy metabolism, and immune function (Clare et al. 2014; Maurice et al. 2015). Advances in molecular techniques, particularly DNA metabarcoding and next‐generation sequencing, now provide robust frameworks for accurately characterizing both diet composition and gut microbial assemblages in wildlife. These approaches have become fundamental to ecological and evolutionary research in Chiroptera (Taberlet et al. 2012; Monterroso et al. 2019; Xiao et al. 2019; Li et al. 2024).

The foraging strategies of insectivorous bats exhibit significant seasonal heterogeneity, primarily driven by the phenology of their arthropod prey assemblages (Gual‐Suárez and Medellín 2021). For example, the prey composition of *Tylonycteris pachypus* undergoes marked seasonal shifts: Diptera dominate as the primary food source in spring, whereas Hymenoptera become the predominant prey during summer (Zhang et al. 2005). As endothermic mammals capable of hibernation, bats' gastrointestinal microbiota also demonstrate notable seasonal dynamics, influenced by environmental variables, physiological states, host genetics, and dietary intake (Whitaker et al. 2004; Lindsay et al. 2020). Prior research indicates that dietary composition serves as a key ecological factor modulating gut microbial consortia, with shifts in nutrient profiles directly inducing structural remodeling and functional diversification of microbial taxa (Muegge et al. 2011; Costantini et al. 2024). Seasonal dietary transitions within chiropteran populations invoke adaptive adjustments in gut microbiota, optimizing host digestion and nutrient extraction from available resources. For instance, *Eptesicus fuscus* transitions to frugivory during periods of prey scarcity, resulting in an increased prevalence of cellulolytic bacterial populations that facilitate carbohydrate breakdown (Clare et al. 2014). Similarly, the dietary shift of *Ia io* from summer to autumn correlates with gut microbial restructuring to augment energetic reserves prior to migration or hibernation (Gong 2022). Nevertheless, investigations into how seasonal dietary shifts mechanistically drive the functional remodeling of gut microbial communities in insectivorous bats remain sparse, particularly regarding the systematic elucidation of microbial metabolic pathway adaptations.

*Myotis chinensis* , a member of the family Vespertilionidae and the genus *Myotis*, is widely distributed across several provinces in China, including Shaanxi, Zhejiang, Jiangsu, Anhui, Sichuan, and Fujian (Liu et al. 2022). Within its range, the bat primarily roosts in large caves, where individuals hang solitarily or in small clusters from the ceiling, and often form mixed‐species colonies with other bats (Sheng 2005). This species exhibits pronounced seasonal phenology: migrating to maternity sites in late March, giving birth in June, and with juveniles achieving independence by mid‐July. The mating season spans September to October, characterized by paired roosting with males positioned dorsally (Shi et al. 2006). As an important insectivorous bat, it plays a vital role in natural pest control and ecosystem stability (Dong et al. 1989). On the basis of the established link between diet and gut microbiota, we propose the following hypotheses: (1) Alterations in dietary composition across seasons are associated with significant restructuring of the gut microbial community and the relative abundance of specific bacterial taxa. (2) The dietary and microbial dynamics are linked to predictable variations in the inferred functional potential of the microbiota, particularly the enrichment of metabolic pathways relevant to nutrient utilization in each season.

To test this hypothesis, we employed DNA metabarcoding and 16S rRNA gene amplicon sequencing, coupled with KEGG‐based functional prediction, to systematically characterize seasonal variations in the diet and gut microbiome of *Myotis chinensis* . This integrated approach was designed to elucidate the adaptive responses of the gut microbiota, including shifts in taxonomic structure and metabolic pathway potential, to fluctuating prey resources. This study elucidates the mechanisms by which *Myotis chinensis* responds to seasonal environmental stresses. Meanwhile, the seasonal shifts in *Myotis chinensis* ' feeding habits indirectly reflect the phenological dynamics of local insect communities, thereby serving as biological indicators for monitoring local insect population fluctuations.

## 2 Methods

### 2.1 Study Area

The study site is located in an abandoned mine cave in Linghou Village, Jinyun County, Lishui City, Zhejiang Province (28°36′–28°39′ N, 120°02′–120°05′ E). The region experiences a typical subtropical monsoon climate characterized by an annual average temperature ranging from 16.5°C to 18.5°C and annual precipitation between 1600 and 1800 mm. The habitat types are complex and diverse, primarily encompassing subtropical evergreen‐deciduous broadleaf mixed forests, rocky mountain shrublands, cultivated drylands, as well as riverine and lacustrine wetland systems. The mine cave is characterized as a limestone karst formation, featuring a south‐facing entrance, and extending approximately 100 m in depth. Inside the cave, both temperature and humidity levels are stable; specifically, the bat roosting area maintains a temperature range of 10°C–15°C with relative humidity consistently exceeding 80%.

### 2.2 Sample Collection and Processing

Field sampling was conducted from March to October 2023, encompassing the spring (from March to early May), early summer (from mid‐May to June), late summer (from July to August), and autumn (from September to October). Following bat capture using harp traps positioned at cave entrances, these were subsequently stored in sterile, contaminant‐free linen bags and transported to the laboratory for further analysis. Detailed external morphological measurements were initially recorded, including key indicators such as gender, weight, body length, and wingspan, to ensure the integrity and scientific value of the samples. Fresh fecal samples were collected immediately upon defecation during handling or from sterile containers. During transport to the laboratory, samples were maintained at −20°C using dry ice. Upon arrival, all samples were transferred to a −80°C ultra‐low temperature freezer for long‐term storage until DNA extraction. Following euthanasia, approximately 200 mg of small intestinal content was aseptically collected during dissection using sterile forceps. After labeling, the samples were rapidly stored in a −80°C ultra‐low temperature freezer to maintain stability at low temperatures. A total of 40 intestinal content samples and 40 fecal samples from *Myotis chinensis* were collected during this study, with 10 samples per season period, and feces were used for analyzing gut microbiota, while small intestinal contents were employed for examining dietary.

### 2.3 Dietary DNA Macrobarcode Analysis

DNA was extracted from the contents of the small intestine using the E.Z.N.A Soil DNA Kit, followed by homogenization and lysis to ensure a high‐quality extraction (Li 2022). The extracted DNA was then subjected to agarose gel electrophoresis and analyzed with a NanoDrop 2000 spectrophotometer to assess its quality, concentration, and purity. Subsequently, it was stored at −20°Cfor future research. To analyze the dietary of *Myotis chinensis* , the mitochondrial cytochrome c oxidase subunit COI gene region was selected as the target fragment. Specific primers LCO1490 (5′‐barcode+GGTCAACAAATCATAAAGATATTGG‐3′) and COI‐CFMRa (5′‐GGWACTAATCAATTTCCAAATCC‐3′) were utilized for PCR amplification. The barcode preceding these primers facilitated sample identification (Kim et al. 2022). The PCR thermal cycling conditions were as follows: initial denaturation at 98°C for 30 s, followed by 25 cycles of denaturation at 98°C for 15 s, annealing at 50°C for 30 s, and extension at 72°C for 30 s, with a final extension at 72°C for 5 min. The amplified products underwent agarose gel electrophoresis; the target fragment was excised, recovered using a gel recovery kit, and quantified with the Quant‐iT PicoGreen dsDNA Assay Kit before proceeding to library construction. DNA fragments were processed utilizing the Illumina TruSeq Nano DNA LT Library Prep Kit for end repair, A‐base addition, and sequencing adapter ligation. Following PCR amplification and purification steps yielded high‐quality sequencing libraries. After quality control measures were implemented, these libraries were sequenced on the NovaSeq 6000 platform employing 2 × 250 bp paired‐end sequencing. The resulting sequencing data were processed using QIIME 2 software to generate high‐quality OTU data. Representative sequences of OTUs were identified through comparisons with GeneBank and BOLD databases (grouping sequences with 97% similarity into the same OTU); low‐abundance OTUs (abundance value below 0.001% of the total sequencing depth across all samples) were subsequently removed to ensure reliable results. Finally, OTU data visualization was performed using *R* software (including Phyloseq, Vegan, ggplot2, DESeq2) to provide scientific evidence regarding dietary habits and ecological adaptation mechanisms of the *Myotis chinensis* .

### 2.4 Analysis of Intestinal Microbial 16S rRNA Amplicons

Extraction of microbial genomic DNA from fecal samples using the E.Z.N.A Soil DNA Kit, and evaluate the quality and purity of the DNA using agarose gel electrophoresis and NanoDrop 2000 spectrophotometry. Confirm sample integrity prior to proceeding with subsequent analyses. Subsequently, PCR amplification of the V3‐V4 hypervariable region of the 16S rRNA gene was performed using primers 338F (5′‐ACTCCTACGGGAGGCAGCAG‐3′) and 806R (5′‐GGACTACHVGGGTWTCTAAT‐3′), followed by library preparation for sequencing. Paired‐end sequencing (PE250) was conducted on the NovaSeq 6000 platform (Teng et al. 2022). The sequencing data underwent quality control filtering, sequence assembly, chimera removal, and construction of an amplicon sequence variant (ASV) feature table to ensure high‐quality valid sequences (grouping sequences with 100% similarity into the same ASV).

### 2.5 Sequencing of Pooled Samples and Differentiated Bioinformatics Analysis

This study investigated community‐level, seasonal associations between diet and gut microbiota by integrating pooled‐sample sequencing with customized bioinformatics. We pooled fecal and intestinal content samples from each season period equally to create representative sequencing libraries. This design captured population‐level seasonal trends, minimized individual noise, and highlighted seasonality as a macroecological driver (Maurice et al. 2015). For dietary and microbiome datasets, we utilized differential sequence clustering techniques tailored to the intrinsic properties of each dataset while fulfilling the specific aims of each analytical module. In dietary metabarcoding, sequences were grouped into OTUs, a conventional method in dietary ecology. This approach effectively reduced sequencing artifacts and addressed the limited scope of arthropod reference databases, allowing accurate identification of prey taxa at the genus or family level, thereby aligning with the central ecological goals of the research (Deagle et al. 2019). Conversely, for gut microbiota 16S rRNA gene sequencing, ASV methodology was applied, providing single‐nucleotide resolution for finer discrimination among closely related bacterial lineages and facilitating reproducibility and comparability of findings across studies (Callahan et al. 2016).

### 2.6 Data Statistics and Analysis

Venn diagrams were created to illustrate the number of shared and unique ASVs across different seasons, focusing on diet and gut microbiota. Statistical analyses were performed on dietary composition, gut microbiota composition, and relative abundance across all samples at various taxonomic levels. A comprehensive analysis of the dietary and gut microbial composition of *Myotis chinensis* across different seasons was conducted. First, α‐diversity indices were calculated, including the Shannon‐Wiener index, Simpson's diversity index, and Pielou's evenness index. Furthermore, principal coordinate analysis (PCoA) was employed to analyze β‐diversity in dietary composition and gut microbial composition across different seasons, all statistical analyses and visualizations were conducted within the R software, utilizing packages including phyloseq, vegan, DESeq2, and ggplot2. To identify key food items and gut microbial species (i.e., key biomarkers) that may vary seasonally in *Myotis chinensis* , linear discriminant analysis effect size (LEfSe, LDA = 4.0) was performed on the relative abundance of food and gut microbial sequences across different seasons using an online platform (Paisenno Gene Cloud) (Segata et al. 2011). Correlations between gut microbiota and dietary composition were established. Ultimately, PICRUSt2 was employed to predict the metabolic functions of the gut microbiome and to conduct analyses of seasonal abundance variations (Dong et al. 2022).

## 3 Results

### 3.1 Sequencing Quality and ASV Analysis

**FIGURE 1:**
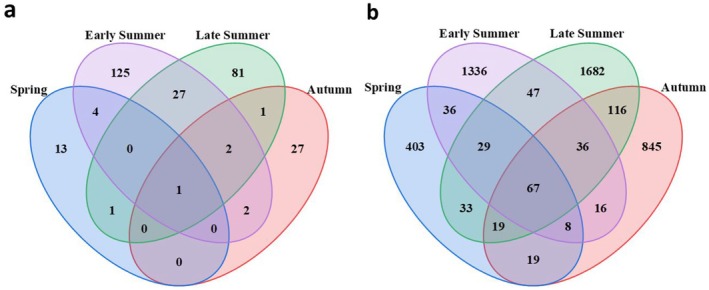
Dietary habits and gut microbiota sequencing results: (a) dietary habits OTU; (b) the number of ASVs in gut microbiota presented in a Venn diagram.

Using Illumina high‐throughput sequencing technology and food DNA macrobarcoding analysis methods, a total of 4,010,329 valid sequences were obtained from 40 intestinal content samples of *Myotis chinensis* , yielding an average of 100,258 valid sequences per sample, with a maximum effective sequence count of 112,123 and a minimum effective sequence count of 96,942. All samples underwent OTU clustering, resulting in the identification of a total of 246 OTUs, with one shared OTU present across all four time periods (Figure 1a). In addition, using 16S rRNA sequencing to analyze the gut microbiota from the fecal samples of *Myotis chinensis* , we obtained a total of 4,541,568 valid sequences, averaging 113,539 valid sequences per sample, with a maximum effective sequence count of 135,898 and a minimum effective sequence count of 99,309. ASV clustering was performed on all samples, leading to the identification of a total of 6082 ASVs. After clustering the ASVs from all four seasons together, it was found that there were 67 ASVs common across all 40 samples. The results were classified into various taxonomic levels: specifically comprising 31 phyla, 92 classes, 173 orders, 328 families, 614 genera; certain ASVs exhibiting high similarity to reference sequences could be further predicted to the species level, collectively encompassing 822 species (Figure 1b).

### 3.2 Dietary Habits and Intestinal Microbial Composition of *Myotis chinensis*

**FIGURE 2:**
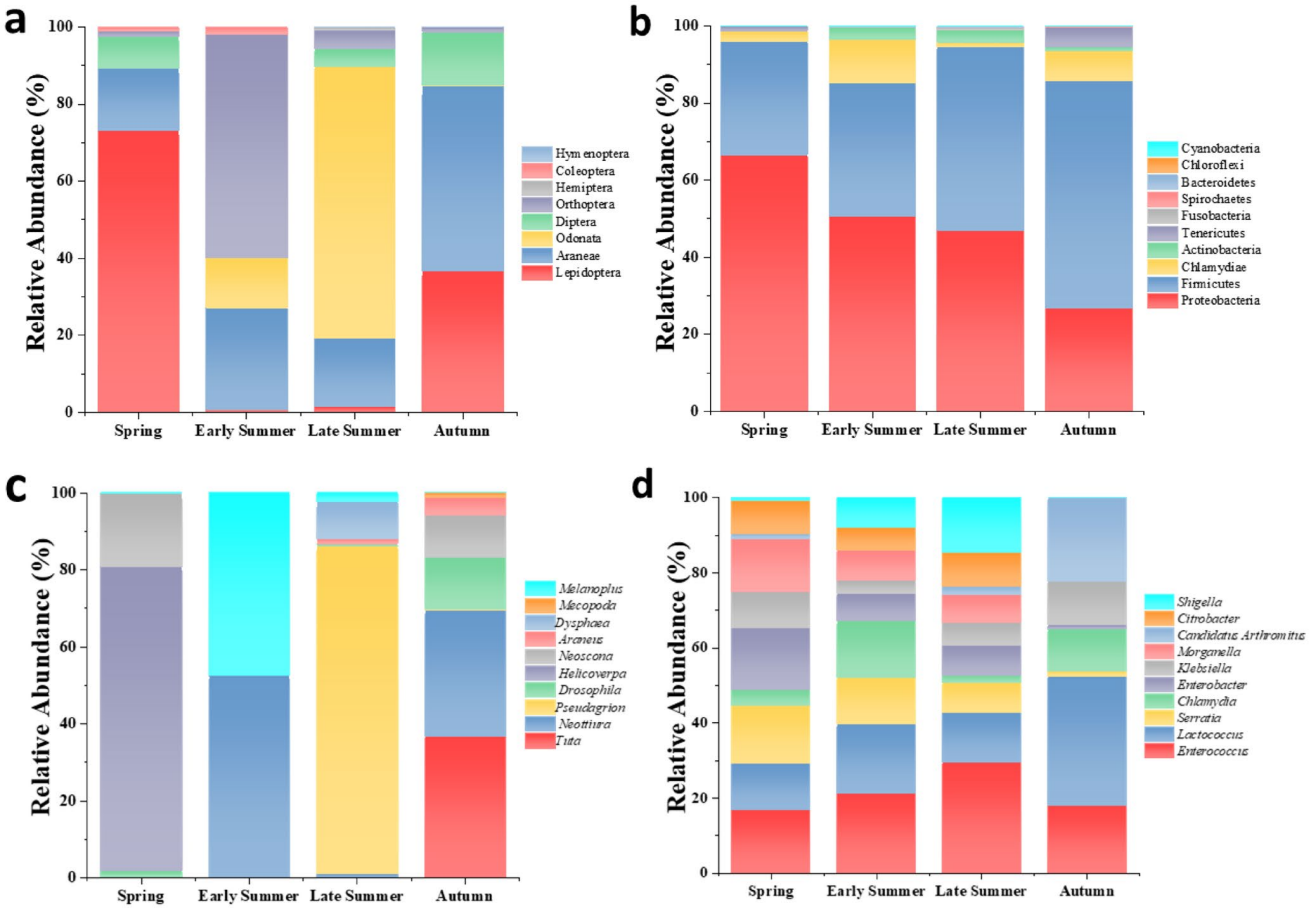
Analysis of dietary habits and gut microbiota composition across different seasons: (a) top eight dietary habits at the order level; (b) top 10 gut microbiota at the phylum level; (c) top 10 dietary habits at the genus level; (d) top 10 gut microbiota at the genus level.

Based on an order‐level analysis, the dietary composition of *Myotis chinensis* encompassed eight insect orders, with their relative abundances exhibiting significant seasonal variations. In spring, Lepidoptera predominates (60.13%), followed by Araneae (13.18%) and Diptera (6.74%). During early summer, the dietary structure underwent notable changes, with Orthoptera emerging as the primary food source (78.19%), supplemented by Odonata. In late summer, Odonata accounted for 79.99%, while Orthoptera (5.47%) and Diptera (5.2%) receded to secondary positions. In autumn, a co‐dominant pattern was observed between Araneae (47.93%) and Lepidoptera (36.57%) (Figure 2a). Genus‐level analysis identified 23 distinct food genus groups, and the dominant genera within the top 10 exhibit clear seasonal specificity. In spring, *Helicoverpa* emerged as the dominant genus with a relative abundance of 79%; in early summer, *Neottiura* (52%) and *Melanoplus* (48%) were co‐dominant; during late summer, *Pseudagrion* constituted 81% of the diet; and in autumn, *Tuta* (37%) and *Neottiura* again appeared prominently at 33% abundance (Figure 2c).

Gut microbiota analysis based on 16S rRNA gene sequencing revealed that the gut microbiota of *Myotis chinensis* comprised 16 phyla, 28 classes, 41 orders, 76 families, and 189 genera. At the phylum level, Proteobacteria (34.2%), Firmicutes (28.7%), and Chlamydiae (18.5%) represent the three predominant microbial groups presented in this species' gut flora. Notably, Proteobacteria dominated during spring through late summer with percentages ranging from 42.1% to 38.6%, whereas Firmicutes increased significantly to account for 35.3% in autumn (Figure 2b). The analysis of genus level indicated that *Enterococcus* served as a core genus throughout all seasons, and its relative abundance demonstrated seasonal fluctuations. It accounted for 10.86% in spring, raised to 16.00% in early summer, peaked at 17.55% in late summer, and decreased to 12.50% in autumn. The secondary dominant genera included *Lactococcus* and *Serratia*, which together constituted the main functional groups of the intestinal microbiota (Figure 2d).

### 3.3 Seasonal Differences in Diet and Gut Microbiota

**FIGURE 3:**
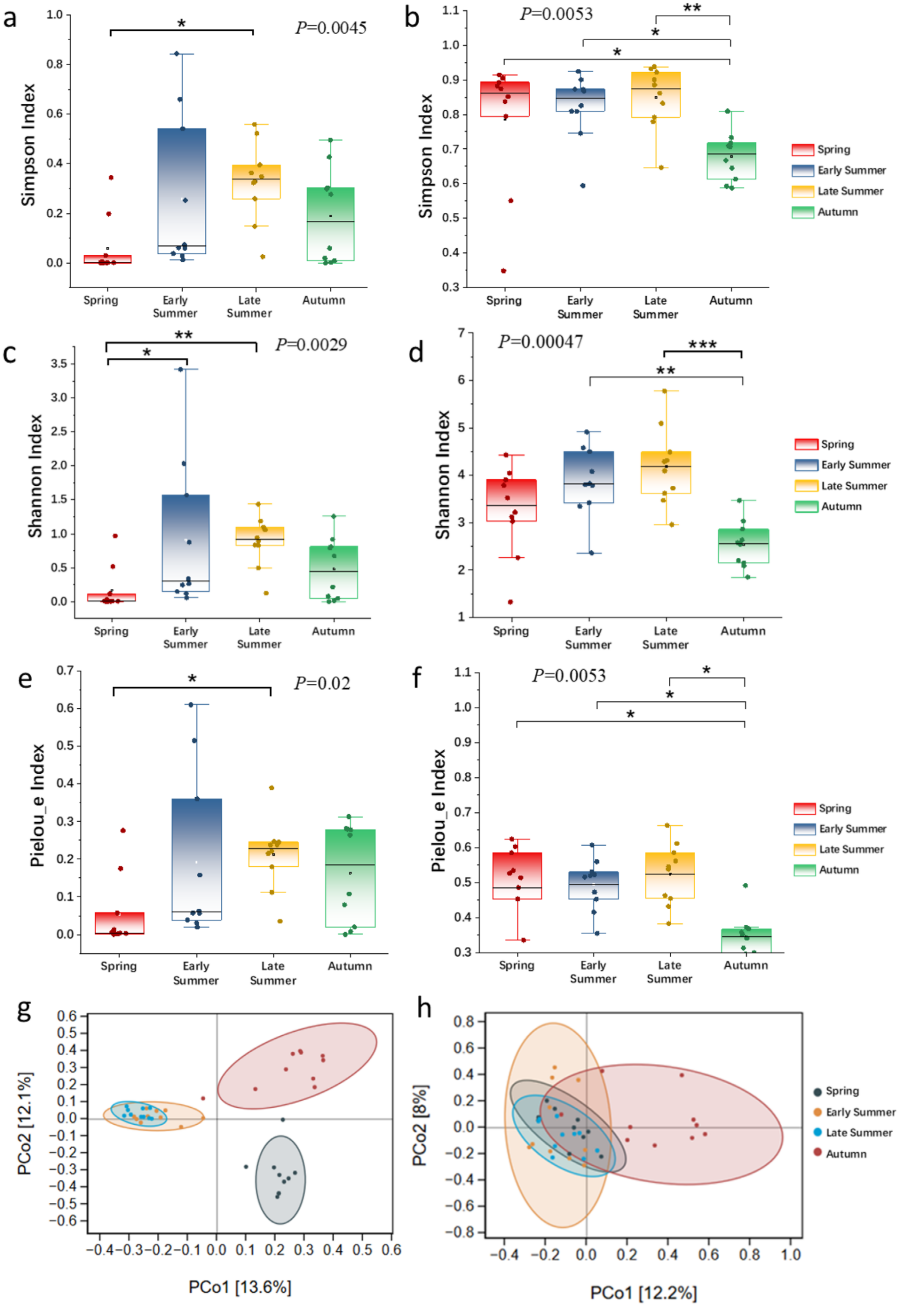
Analysis of dietary habits and gut microbial diversity across different seasons: (a) Simpson index of dietary habit; (b) Simpson index of gut microbiota; (c) Pielou_e index of dietary habit; (d) Pielou_e index of gut microbiota; (e) Shannon‐Wiener index of dietary habit; (f) Shannon‐Wiener index of gut microbiota; (g) PCoA analysis of dietary habit; (h) PCoA analysis of gut microbiota. * Indicates statistical significance levels: *p < 0.05, **p < 0.01, ***p < 0.001.

Dietary α‐diversity analysis revealed significant differences among the three groups during the early summer season. The early summer group exhibited the highest diversity, as indicated by the Shannon index, and demonstrated a more even species distribution, reflected in the Pielou_e index, compared to the other groups. Conversely, while the spring group showed the highest diversity according to Simpson's index, it recorded both lower overall diversity and a more uneven species distribution in terms of both Shannon and Pielou_e indices. The late summer and autumn groups displayed relatively similar performance across all three diversity indices, with no statistically significant differences observed (Figure 3). Principal coordinate analysis (PCoA) results suggested that dietary habits of *Myotis chinensis* across different seasons overlapped to some extent due to similarities in food composition, while autumn samples were notably more dispersed. The dispersion indicated greater variability in food composition species and abundance for *Myotis chinensis* during autumn. In contrast, spring and late summer samples exhibited tighter spatial clustering, suggesting higher dietary similarity between these two groups with smaller inter‐group differences (Figure 3g).

The results of α‐diversity calculations for gut microbiota indicated that both Shannon‐Wiener index and Simpson's diversity index, as well as Pielou_e evenness index, were significantly lower for gut microbiota diversity in *Myotis chinensis* during autumn compared to other seasonal periods (Figure 3). Additionally, PCoA results illustrated that gut microbial compositions of *Myotis chinensis* from various seasons shared overlapping. However, the samples in autumn displayed a more dispersed distribution pattern, indicating greater variability in gut microbial species and abundance among autumn samples. In contrast, samples from spring and late summer exhibited reduced inter‐sample differences leading to a more clustered spatial distribution (Figure 3h).

**FIGURE 4:**
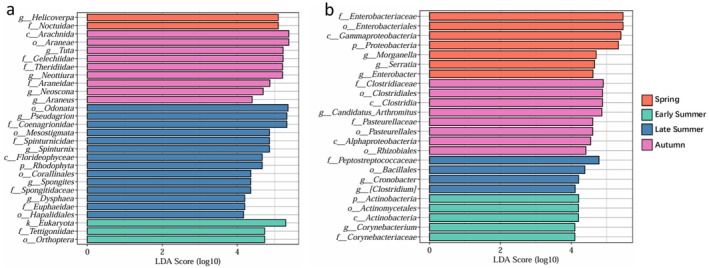
LEfSe analysis of dietary habits and sequence abundance of gut microbiota of *Myotis chinensis* across different season periods: (a) LEfSe analysis of dietary habits; (b) LEfSe analysis of gut microbiota. The vertical axis represented taxonomic units that exhibited significant differences among seasons, while the horizontal axis displayed the logarithmic scores of the corresponding taxonomic units derived from linear discriminant analysis (LDA), LDA > 4, *p* < 0.05.

LEfSe analysis clarified the biomarker taxonomic groups that showed significant differences and enrichment in different seasons. Regarding dietary composition, spring was characterized by the family Noctuidae and the genus *Helicoverpa*; early summer was dominated by the family Tettigoniidae; late summer exhibited enrichment of the families Coenagrionidae, Spinturnicidae, and Spongitidaceae; and autumn was distinguished by the families Gelechiidae and Araneidae (Figure 4a). Within the gut microbiota, spring displayed significant enrichment of the family Enterobacteriaceae, including the genera *Morganella*, *Serratia*, and *Enterobacter*; early summer was characterized by the family Corynebacteriaceae; late summer showed enrichment of the family Peptostreptococcaceae and the genus *Cronobacter*; and autumn was dominated by the families Clostridiaceae and Pasteurellaceae, among others (Figure 4b). The observed covariation between prey availability and microbiota dynamics suggests that the host may modulate intestinal microbial functions via dietary adjustments to cope with seasonal energy requirements and environmental challenges.

### 3.4 Correlation Analysis Between Dietary and Gut Microbiota Composition

**FIGURE 5:**
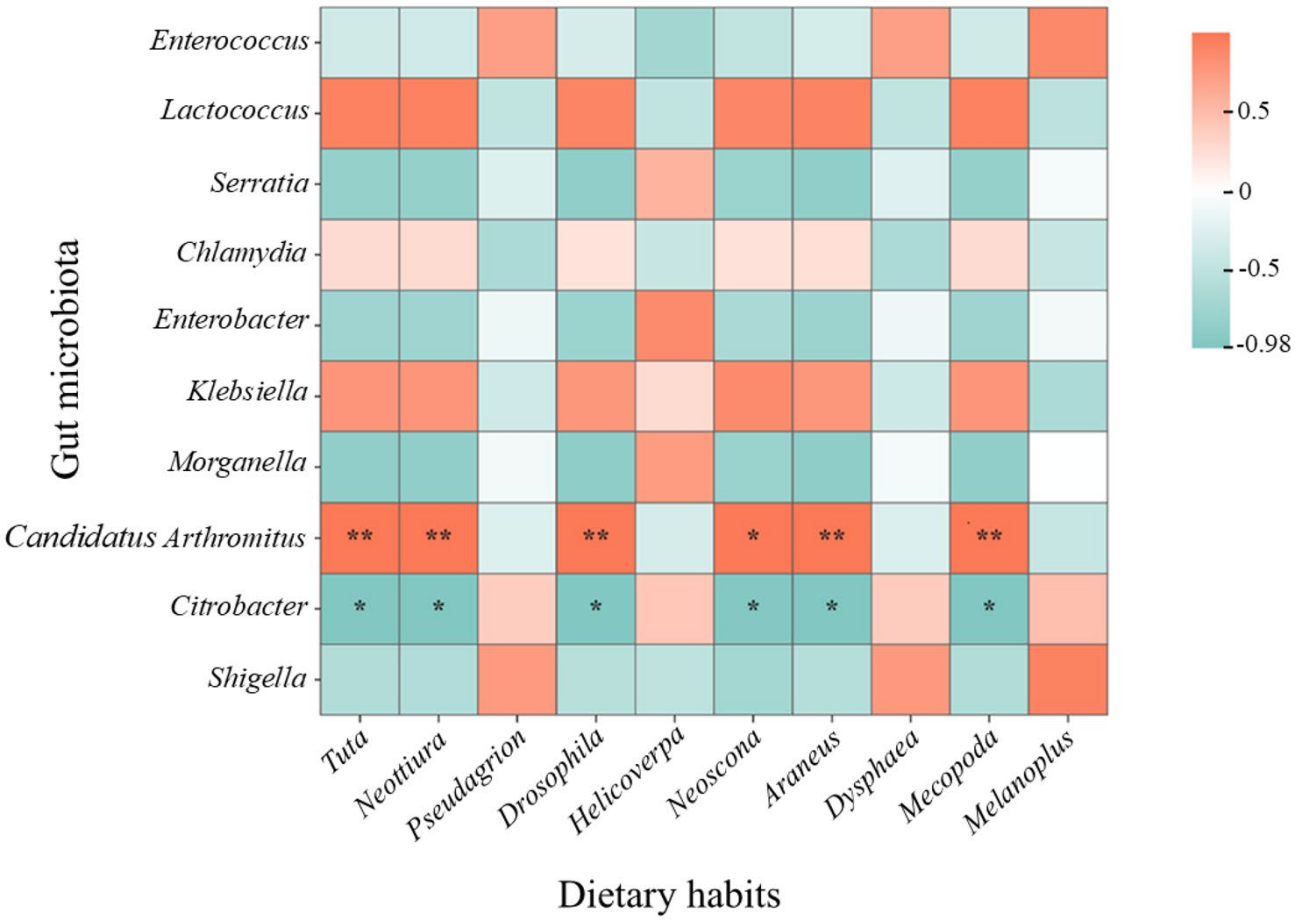
Heatmap of correlation between top ten abundant gut microbial genera (Y‐axis) and dietary habits (X‐axis). The color gradient ranged from blue (indicating a negative correlation) to red (indicating a positive correlation). * represents the level of correlation: *p < 0.05, **p < 0.01.

Spearman correlation analysis demonstrated significant correlations between specific microbial genera and dietary components: *Enterococcus* exhibited a positive correlation with *Helicoverpa* (*p* < 0.05), *Lactococcus* showed a positive association with *Tuta*, while *Shigella* correlated positively with *Melanoplus*. Notably, *Serratia* displayed a significant negative correlation with *Tuta*, *Neottiura*, and *Drosophila* (*p* < 0.05), alongside *Morganella*'s significant negative correlation with *Drosophila* (*p* < 0.05). At an elevated significance threshold (*p* < 0.01), *Candidatus arthromitus* exhibited a statistically significant positive association with *Tuta*, *Neottiura*, and *Drosophila*, whereas *Citrobacter* demonstrated a significant negative correlation with these taxa (*p* < 0.05) (Figure 5).

The correlation results obtained in this study supported the genus‐specific differences identified through LEfSe analysis, highlighting a significant association between seasonally distinct microbial community structures and dietary habits. These findings suggested that *Myotis chinensis* had developed an effective physiological adaptation mechanism via the synergistic modulation of dietary selection and gut microbiota composition. The adaptive strategy might represent a crucial evolutionary approach for this species to manage seasonal environmental stressors and fluctuations in ecological resources.

### 3.5 Analysis of Seasonal Differences in Predicted Gut Microbial Function

**FIGURE 6:**
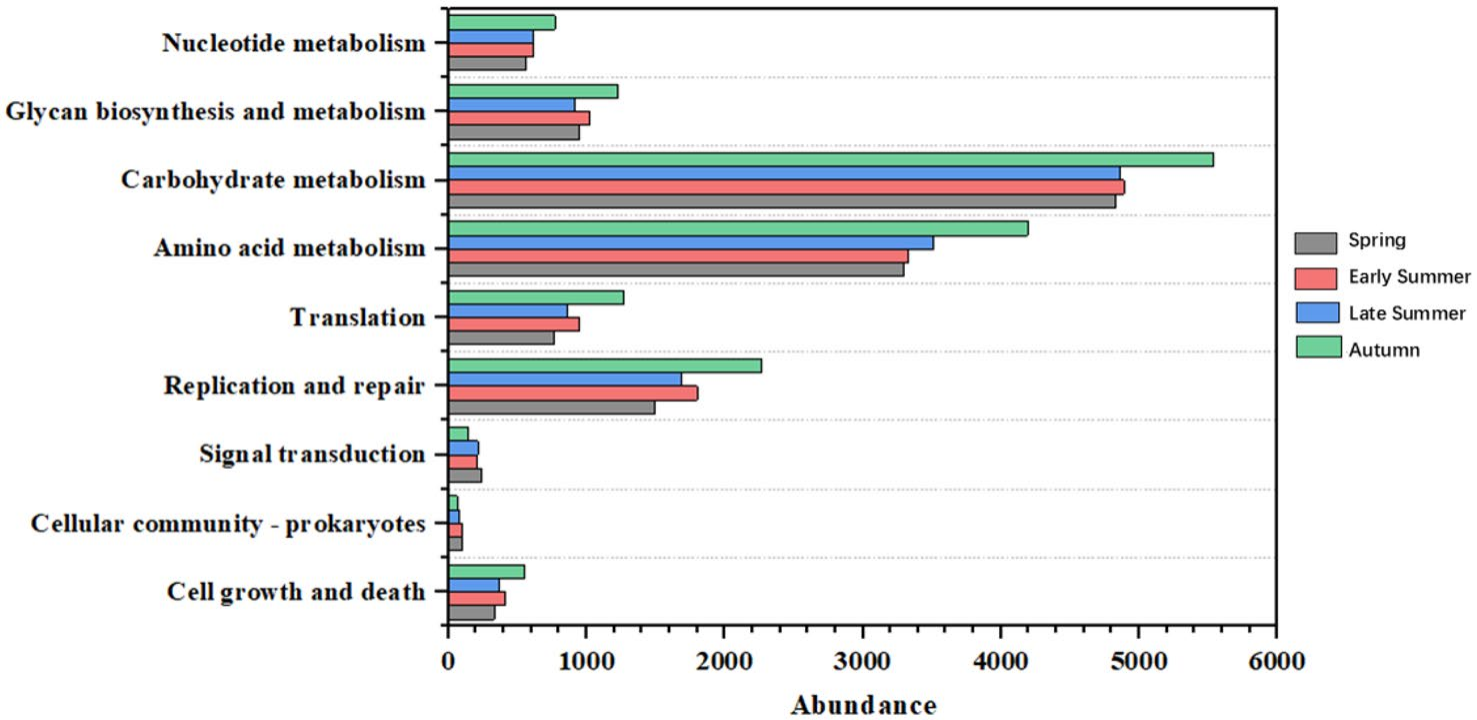
Seasonal variations in the abundance of secondary functional metabolic pathways of gut microbiota predicted by KEGG.

The results of functional predictions from PICRUSt2 indicated significant seasonal variations in certain pathways among the predicted secondary metabolic pathways of intestinal microorganisms. Most pathways exhibited a marked increase in abundance during autumn, including carbohydrate metabolism, amino acid metabolism, translation, replication, and repair pathways. Conversely, only a limited number of pathways demonstrated higher abundance during spring, such as those related to cellular community‐prokaryotes and signal transduction (Figure 6).

**FIGURE 7:**
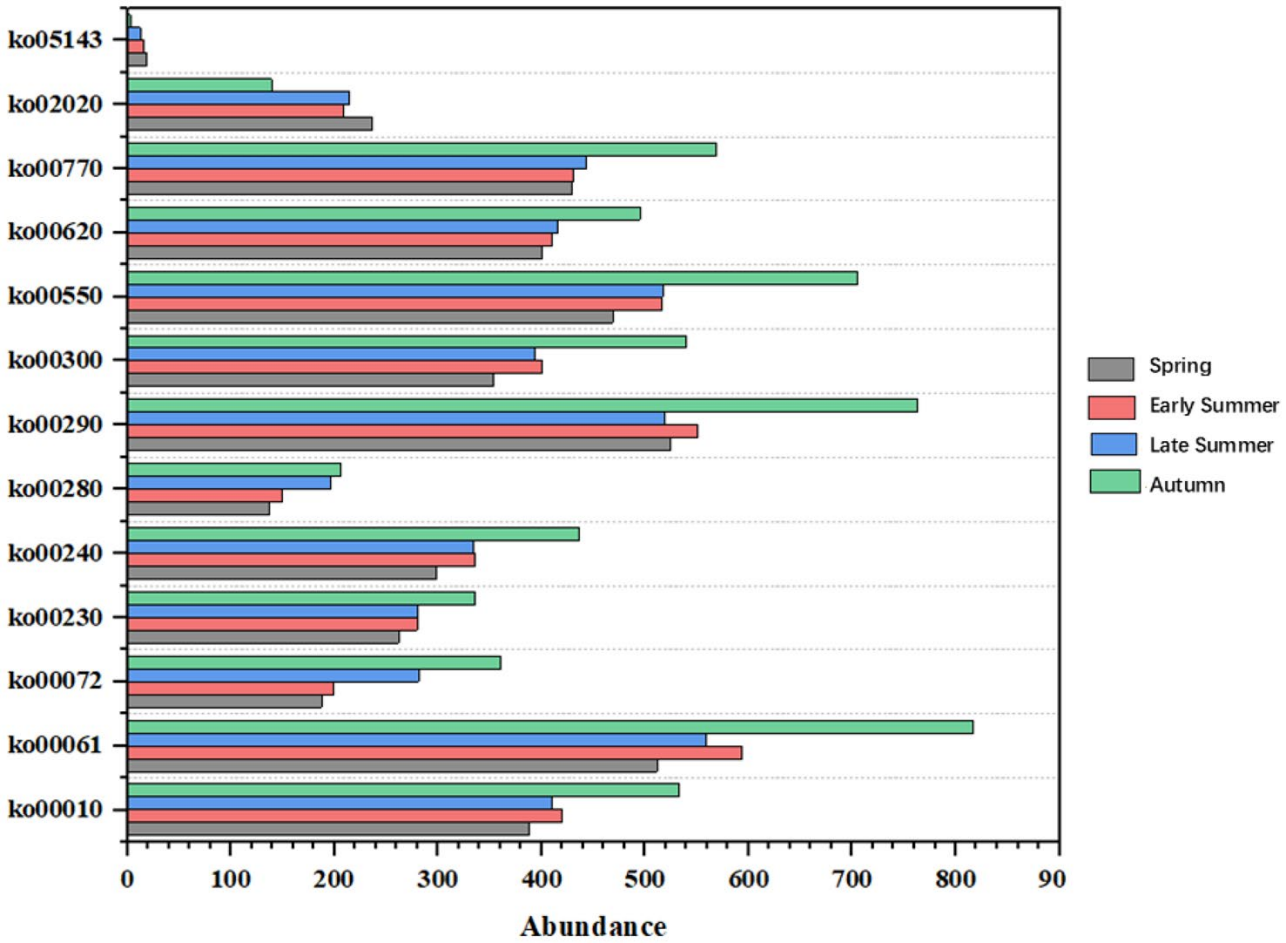
Abundance of gut microbial tertiary functional metabolic pathways in *Myotis chinensis* in different seasons.

As illustrated in Figure 7, several pathways within the predicted tertiary functional metabolic networks of gut microbiota displayed significant seasonal differences. The abundance of most functional pathways significantly increased in autumn, particularly sphingolipid metabolism (ko00550), protein digestion and absorption (ko00290), and fatty acid biosynthesis (ko00061). Furthermore, there was a substantial rise in the biosynthesis of coenzymes and other terpenoid‐quinones (ko00770), phenylalanine, tyrosine, and tryptophan biosynthesis (ko00620), steroid hormone synthesis (ko00072), as well as metabolic processes such as tyrosine metabolism (ko00300) and purine metabolism (ko00240) during autumn. However, for some specific pathways—such as malaria pathway (ko05143) and fructose and mannose metabolism pathway (ko02020)—the abundance observed in autumn was significantly lower than that recorded in other seasons. These findings suggest that certain intestinal microbial functions within *Myotis chinensis* exhibit seasonal variability to adapt to changes associated with different seasons and dietary shifts.

## 4 Discussion

### 4.1 Diet‐Driven Seasonal Reshaping of Gut Microbial Communities

The dietary habits of *Myotis chinensis* exhibited significant seasonal dynamics, which drove adaptive reshaping of its gut microbiota. In spring, bats primarily consumed Lepidoptera, characterized by relatively low protein content. However, during the summer, their diet shifted toward Orthoptera and Odonata, both of which possessed significantly higher protein content (Churchward‐Venne et al. 2017). This alteration in nutrient input is likely linked to the succession within the microbial community: the relative abundance of the Firmicutes phylum markedly increased during summer. Members such as *Lactococcus* accelerate protein breakdown and promote energy conversion by secreting proteases and short‐chain fatty acid synthase. Furthermore, short‐chain fatty acids serve as a key energy source, playing a direct role in the host's energy metabolism and supporting the high metabolic demands during the bat breeding season and lactation period (Gaudu et al. 2019; Lee et al. 2025). Additionally, *Enterobacter—a* genus within the Proteobacteria phylum known for its amino acid transport functions—also showed significant enrichment during summer, further enhancing protein utilization efficiency. In contrast to these findings, Lee and Kelleher reported that lactating bats increased their dietary diversity by incorporating Diptera and selecting high‐protein foods to meet their nutritional requirements effectively (Lee and Kelleher 2016). This discrepancy may not represent a contradiction but rather reflects differences in optimal foraging strategies under distinct ecological contexts. In our study, the summer habitat of *Myotis chinensis* likely offered access to “high‐quality, high‐biomass” resources such as Orthoptera and Odonata. In such a scenario, “specialized predation” on these protein‐rich, large‐bodied prey represents the most efficient strategy for meeting energy demands. Conversely, in the environment examined by Lee and Kelleher (2016), Diptera may have been relatively more abundant or easier to capture, making a “diversification strategy” —broadening the dietary spectrum by incorporating Diptera—an effective approach to ensuring nutritional adequacy. Together, these findings illustrate that the high energy demands of lactation can be met through different pathways, the choice of which depends fundamentally on the local prey community structure and availability. Furthermore, Whitby suggested that though Orthoptera were rich in protein content, most species were diurnal with few nocturnal species active on the ground, which made it hard for bats to hunt them (Whitby et al. 2020). Moreover, Laurent found that compared to non‐lactating bats, lactating bats exhibited a higher relative abundance of Actinobacteria and a lower relative abundance of Firmicutes (Tarnaud 2006). This microbial profile may facilitate increased fat metabolism and reduced fat storage, thereby enhancing offspring survival rates (Hamady et al. 2008). These studies underscore the significance of the host's physiological state and suggest that future research should correlate dietary habits, the microbiome, and precise reproductive status data at the individual level.

As autumn approached, the composition of prey shifted to Araneae (relative abundance 47.9%), whose exoskeletons were rich in chitin. This shift drived gut microbial functions toward chitin degradation and lipid storage (Machałowski et al. 2019). Concurrently, functional predictions from PICRUSt2 indicated that lipid metabolism pathways (e.g., ko00061 fatty acid synthesis) were significantly upregulated in autumn (*p* < 0.05), accompanied by a synchronous increase in the abundance of *Butyrivibrio*, a genus associated with fat storage within the Firmicutes phylum (Delphine et al. 2007). These findings suggested that microorganisms employed a dual ‘degradation‐conversion’ mechanism to transform chitin‐derived carbon sources into lipid reserves, providing energy storage for hibernation‐related metabolic deficits. The synergistic response among diet, microbiota, and metabolic function highlighted the gut microbiome's role as an ecological bridge enabling *Myotis chinensis* to adapt to seasonal environmental stressors.

### 4.2 Functional Adaptability Supports Environment Adaptation

Lactation represents the most energy‐intensive activity during the reproductive process of female mammals (Vanslambrouck et al. 2021). Bats are the only mammals capable of true flight. During lactation, females face exceptionally high energy demands‐not only to produce milk but also to carry their offspring during flight, which requires extraordinary metabolic expenditure (Kunz et al. 1995; Mclean and Speakman 2002). Therefore, the reproductive status of the host is a key intrinsic factor in explaining its energy metabolism and potential microbial functions. The functional adaptability of gut microbiota in *Myotis chinensis* serves as a fundamental mechanism for coping with seasonal environmental challenges. Summer, particularly late summer, represents the peak period for reproduction and lactation, accompanied by a surge in energy demands. At this time, prey composition is predominantly comprised of high‐protein Odonata species, leading to a significant enhancement of amino acid metabolic functions within the gut microbiota. PICRUSt2 analyses revealed that expression levels of the valine/leucine/isoleucine degradation pathway (ko00280) were significantly upregulated compared to those observed in spring. Meanwhile, *Enterobacteriaceae* from the Proteobacteria phylum and *Lactococcus* from the Firmicutes phylum contributed to ketone acid and ATP production through protein decomposition from prey items, may thereby contribute to meeting the energy demands of bats during extended flight and lactation (Famularo et al. 2005). Furthermore, sulfur‐containing amino acids, such as methionine, which are abundant in Odonata—can activate microbial sulfur metabolism pathways that promote glutathione synthesis, and alleviate oxidative stress experienced during flight, which help maintain physiological homeostasis throughout the reproductive period (Siddiqui et al. 2024). In autumn, the diet of prey shifted to Araneae, which have a lipid content ranging from 18% to 25%. Concurrently, gut microbial function transitions into an energy storage mode. The relative abundance of the phylum Firmicutes increases to 66.8%, with core gen era such as Butyrivibrio facilitating the conversion of prey lipids into short‐chain fatty acids through the fatty acid synthesis pathway (ko00061). Additionally, these microbes activate the ketone body metabolic pathway (ko00072) to convert acetyl‐CoA into β‐hydroxybutyrate, thereby providing sustained energy for hibernation (Immanuel et al. 2012; Liu et al. 2024).

### 4.3 Limitations of the Study and Future Prospects

This study integrated dietary metabarcoding with gut microbiome sequencing, revealing a seasonal pattern of synergistic variation in diet, microbial community structure, and predicted function in the Chinese long‐eared bat. However, several limitations should be acknowledged and pointed new directions for the future research. First, to directly assess the dominant role of season as a macro‐ecological driver, we employed a pooled‐sample strategy at the population level. Although individual sex was recorded, this design captured population‐level seasonal trends and did not permit statistical disentanglement of how intrinsic host factors (e.g., sex, lactation status) interact with seasonal diet to shape the gut microbiota. Future longitudinal studies of marked individuals would allow a more precise quantification of the relative contributions of these factors. Second, microbial functional inference relied on PICRUSt2 predictions from 16S rRNA gene data, which reflected functional potential rather than in vivo metabolic activity. Direct validation using multi‐omics approaches—such as metagenomics, metatranscriptomics, or metabolomics—is needed to confirm the specific roles of the predicted metabolic pathways in host adaptation. Third, sampling was limited to March–October and did not include the winter hibernation period. Consequently, a full annual perspective on diet‐microbiota dynamics is incomplete. Collecting samples across pre‐hibernation, hibernation, and post‐hibernation phases would help clarify how gut microbes support extreme energy conservation and reactivation during hibernation.

## 5 Conclusions

This study systematically elucidated the synergistic adaptive mechanisms between the seasonal dietary fluctuations of the *Myotis chinensis* and the structural and functional dynamics of gut microbiome, providing robust support for the proposed hypothesis. The findings revealed that the dietary composition of the *Myotis chinensis* undergoes significant seasonal variation: Lepidopteran insects predominate in spring, Orthopterans and Odonatans in summer, and Arachnids in autumn. Correspondingly, gut microbiota diversity exhibited a dynamic pattern, peaking in late summer and reaching its nadir in autumn, with microbial community structure closely linked to prey resource composition across seasons. Functional predictive analyses indicated that during summer, the gut microbiome upregulates carbohydrate and amino acid metabolic pathways to meet the host's heightened energetic requirements during reproduction. In autumn, microbial pathways related to lipid metabolism and hibernation are enriched, facilitating energy storage for overwintering. This ‘diet‐microbiota‐metabolic function’ interconnected adjustment underscored the microbiome's pivotal role as an intermediary in the host's adaptation to seasonal environmental stressors.

These results supported our proposed hypotheses: the seasonal dietary shift from Lepidoptera to Orthoptera/Odonata dominance was accompanied by significant alterations in gut microbial community structure and in the relative abundance of specific bacterial genera (e.g., *Lactobacillus*, *Enterobacter*); and these changes were correlated with a predictable enrichment of specific microbial metabolic pathways (e.g., amino acid degradation in summer and fatty acid synthesis in autumn), as inferred from genomic potential. Together, these findings indicate that diet‐driven restructuring of the gut microbiota and its predicted metabolic capacity were integral to the seasonal adaptation of *Myotis chinensis* .

## Author Contributions

**Ruohan Xiong:** conceptualization (equal), formal analysis (equal), methodology (equal), visualization (equal), writing – original draft (equal), writing – review and editing (equal). **Jing Li:** conceptualization (equal), data curation (equal), formal analysis (equal), methodology (equal), writing – review and editing (equal). **Ke Wang:** data curation (equal), validation (equal). **Hongying Kong:** data curation (equal), formal analysis (equal), visualization (equal). **Zhengyu Dai:** data curation (equal), formal analysis (equal), visualization (equal). **Yanni Wang:** funding acquisition (equal), resources (equal), supervision (equal). **Rongquan zheng:** funding acquisition (equal), resources (equal), supervision (equal).

## Funding

This work was supported by the National Natural Science Foundation of China, 31200323. Zhejiang Provincial Natural Science Foundation, LJHSD26C040001. Zhejiang Province Pangolin Conservation and Rescue Initiative for Rare and Endangered Wild Fauna, KYH34424024.

## Ethics Statement

The experimental protocol involving animals for this research adheres to established standards for animal welfare and ethical considerations. It has received approval from the Animal Ethics Committee at Zhejiang Normal University, with the institutional review approval number: ZSDW2025044.

## Conflicts of Interest

The authors declare no conflicts of interest.

## Data Availability

The dietary and gut microbiota sequencing datasets for the *Myotis chinensis* obtained in this research have been deposited in the Sequence Read Archive (SRA) under accession number PRJNA1327667. Comprehensive metadata can be accessed at https://www.ncbi.nlm.nih.gov/bioproject/PRJNA1327667. No specialized bioinformatics pipelines or custom scripts were utilized; the analytical tools employed operated with default settings.
